# Understanding the Relationship between Surface Quality and Chip Morphology under Sustainable Cutting Environments

**DOI:** 10.3390/ma17081826

**Published:** 2024-04-16

**Authors:** Mustafa Günay, Mehmet Erdi Korkmaz

**Affiliations:** Department of Mechanical Engineering, Faculty of Engineering, Karabük University, 78050 Karabük, Turkey; mgunay@karabuk.edu.tr

**Keywords:** PH13-8Mo, milling, surface morphology, MQL, cryogenic, chip formation

## Abstract

Although chip morphology changes according to the machining method and related cutting parameters, chip formation affects the quality of the machined surface. In this context, it is very important to understand the relationship between chip morphology and surface quality, especially in materials that are difficult to machine. In the presented study, the changes in chip morphology, surface morphology, and surface quality criteria (Ra and Rz) that occurred during the milling of precipitation-hardened steel in different cutting environments were analyzed. Milling experiments were carried out in dry, MQL (minimum quantity lubrication), nano-MQL (graphene), nano-MQL (hBN), Cryo, and Cryo-MQL environments using TiAlN-coated inserts and three different cutting speeds and feed rates. While the highest values in terms of Ra and Rz were measured in dry machining, the minimum values were obtained in a nano-MQL (hBN) cutting environment. Due to the lubrication and low friction provided by the MQL cutting environment, chips were formed in thinner segmented forms. This formation reduced the chip curve radius and thus provided a more stable surface morphology. On the other hand, Cryo-ambient gas could not effectively leak into the cutting zone due to the intermittent cutting process, but it increased the brittleness of the chips with the cooling effect and provided a similar surface morphology. The values of minimum Ra and Rz were obtained as 0.304 mm and 1.825 mm, respectively, at a 60 m/min cutting speed and 0.04 mm/rev feed. Consequently, the use of nano-MQL cutting medium is seriously recommended in terms of surface quality in milling operations of difficult-to-machine materials.

## 1. Introduction

PH13-8Mo steel, a material in the precipitation-hardened (PH) stainless steel group, exhibits high strength, good toughness, and high endurance to general corrosion and stress–corrosion cracking [1,2]. This material maintains its mechanical properties considerably even under harsh environmental conditions [3]. On the other side, poor thermal conductivity, extraordinary mechanical properties, ductility, and work hardening make the machinability of materials such as stainless steel difficult [4,5]. For these materials, selecting factors, for example, process parameters, cutting environment, and tool properties, are vital for optimizing machinability indicators such as surface properties, machining force, tool life, and consumed energy [6].

**Figure 1:**
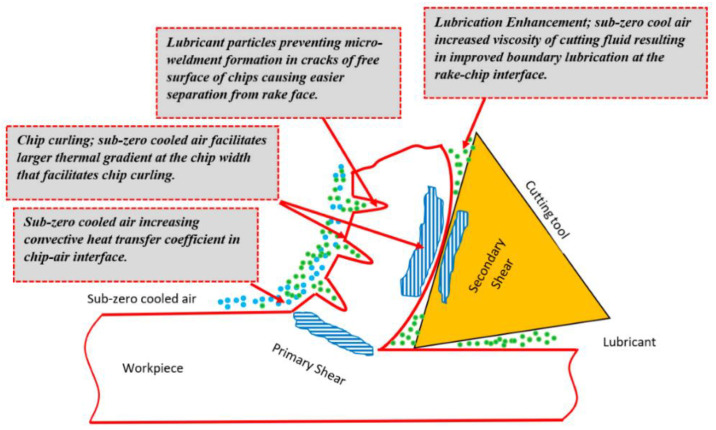
Schematic of MQL mechanism [17].

In machining methods with complex cutting mechanics, such as milling, the above-mentioned process becomes even more critical [7,8]. The side and back surfaces of the tool create a large frictional force in up-milling [9,10]. This force not only raises the cutting force and temperature, as well as causing material deformation, but also exacerbates tool wear and worsens the machined surface quality [11,12]. The cutting force is an essential machinability indicator for determining the loads and stress on tool and power consumption, and tool wear and surface integrity deteriorate when high [6,13]. At this point, MQL and nano-MQL cutting environments provide significant positive benefits on machinability outputs such as cutting forces, specific cutting energy, and tool wear compared to dry cutting in turning or milling applications [14,15,16]. This aligns with the nomenclature of the procedure, indicating that it pertains to the MQL method. A nozzle, which is part of an MQL system, is responsible for directing pressurized air into the specific region where the workpiece and the tool are situated, as shown in Figure 1 [17]. On the other hand, some studies stated that during milling in different cryogenic temperatures, cutting forces increase due to the increase in material microhardness, and surface roughness decreases due to the chip formation that varies according to thermo-mechanical combination impact [18,19,20].

Surface roughness is another important indicator, and different roughness patterns occur depending on the parameters of the machining methods [21,22]. In this context, surface roughness is a critical pointer of machined part quality, significantly affecting the components’ operating performance and mechanical properties, namely fatigue and corrosion resistance [23,24]. In particular, surface quality increases due to the narrowing of the tool adhesion zone, due to the lubrication formed in MQL and hybrid cutting environments [25,26]. At the same time, it was pointed out that the effect of the cutting environment may vary depending on the milling technique (up or down), and it has been emphasized that there is a need for optimization of cutting environments and cutting parameters for cleaner machining [27]. However, the surface quality deteriorates due to tool damage such as microfractures and chipping that occur on the cutting tool in the cryogenic environment [28,29]. On the other hand, it is emphasized that MQL + Cryo contributes to sustainable machining for outputs such as cutting force and surface quality in drilling applications [30]. As can be understood from the research in the literature, the effects of MQL, nano-MQL, cryogenic, or hybrid applications formed by combining them on the machinability indicators vary [31]. In this context, optimizing the cutting tool and cutting conditions according to the cutting environment is very important for sustainable machining [32,33]. In addition to these advantages, recycling of chips is also more economical since the chips obtained after sustainable machining are almost oil-free. Despite all these advantages, studies are being carried out to increase the performance of the MQL method, which has been compared with other cooling methods and dry machining [34].

In recent years, MQL, cryogenic cooling, and hybrid cooling/lubrication techniques have been conducted to increase processing efficiency and provide sustainable machining [35,36,37,38]. Sivaiah and Chakradhar [39] performed a turning process for AISI 630 stainless steel with dry, liquid cooling, cryogenic, and MQL methods at different cutting depths and examined tool wear and chip forms. In this paper, the best surface quality was obtained in cryogenic cooling, and the best surface quality after cryogenic cooling was obtained in the MQL method. As in surface quality, the least wear in free surface wear was obtained with cryogenic cooling, and the least wear values after cryogenic cooling were obtained in the MQL method. In their experimental study, Patole and Kulkarni [40] examined the turning of AISI 4340 material at different feed, flow rate, depth of cut, and cutting tip radius values using liquid cooling and the MWCNT (multi-walled carbon nanotube)-added MQL method. It has been stated that when applied correctly, the nano-MQL method provides serious improvements in cutting forces and surface quality and can be used as an alternative to liquid cooling. Marques et al. [41] performed a turning process for the superalloy Inconel 718 alloy with the MQL method by adding graphite and MoS2 into vegetable cutting oil and determined that the addition of graphite and MoS2 reduced the surface roughness. In their study, Paturi et al. [42] conducted a comparison between the MQL and nano-MQL procedures. The MQL method involved the addition of micro-sized WS2 (Tungsten disulfide) to the oil at a weight rate of 0.5% during the turning process of Inconel 718 alloy. The investigation revealed that the incorporation of WS2 into MQL oil resulted in a notable enhancement of surface quality, with an observed improvement of up to 35%. In their work, Rahim et al. [43] conducted a turning investigation on AISI 1045 material using both dry and MQL methods. The researchers employed synthetic ester as the oil in their experiment. The MQL approach has been found to substantially decrease cutting forces, cutting temperature, and tool–chip contact distance in comparison to dry machining. In their study, Kedare et al. [44] conducted a comparison between MQL and liquid cooling methods in the end milling of mold steel. They found that the MQL approach resulted in a significant improvement in surface quality, with an increase of up to 27%. The turning research conducted by Joshi et al. [45] studied the machining of the Inconel600 alloy and employed the dry, MQL, and nano Al_2_O_3_ (aluminum oxide) enhanced MQL technique. The nano-MQL approach has been reported to yield superior surface quality values compared to alternative cooling methods.

In light of the literature studies, lots of difficult-to-cut materials show better machinability in cryogenic and hybrid cryogenic MQL environments under different cutting conditions. However, PH13-8Mo steel distinguishes itself from other difficult-to-cut materials due to its exceptional strength, excellent toughness, and remarkable resistance to both general corrosion and stress–corrosion cracking. For that reason, cryogenic cooling may not be effective in improving this material’s machinability. The machinability of PH13-8Mo steel commonly used in the aircraft and nuclear industries poses significant challenges, as seen by the scarcity of research conducted on this particular material. Moreover, the combination of nanoparticle-added MQL (nano-MQL) and cryogenic cooling has not been studied yet. In this context, as a novelty of this study, the variation in surface roughness and chip morphology were comprehensively investigated during up-milling in sustainable cutting environments, namely, dry, MQL, nano-MQL, Cryo, and Cryo-MQL conditions. The originality of this study is to understand the detailed relationship between surface integrity and chip morphology in the cleaner milling of PH13-8Mo steel under sustainable cutting environments. This study will make an important contribution to sustainable machining by filling the gap in the literature.

## 2. Apparatus and Methods

### 2.1. Workpiece and Cutting Equipment

In milling operations, PH13-8Mo steel (Bircelik, İstanbul, Turkey) was used as the workpiece, which is the chemical compound of the 110 × 100 × 50 mm workpiece material given in Table 1. The tensile and yield strength, elongation, hardness, and thermal conductivity of PH13-8Mo steel are 1400 MPa, 1300 MPa, 14%, 42 HRC, and 14 W/m-K, respectively. The workpiece material is commonly used in producing valves and shafts operating under various chemical liquids, and substantial components in the aerospace and defense industries, due to its excellent compatibility of superior corrosion and mechanical strength.

TiAlN-coated inserts and a tool holder with a 90° approach angle were used. Up-milling tests were carried out at three different cutting speeds (Vc), three different feed rates (f), and at a constant axial depth, which are the control factors recommended by the cutting insert company (Table 2) under six different cutting environments by full factorial design.

In the milling experiments, the arithmetical roughness value (Ra) and the maximum height of profile (Rz) were measured with a cut-off of 0.8 mm and a sampling length of 4 mm by using the Mahr Perthometer M300 (Mahr, Göttingen, Germany). The roughness measurements were made from three locations, and the Ra was evaluated by averaging the measurements. Moreover, the chips after the machining under each condition were analyzed in CARL ZEISS ULTRA PLUS GEMINI-branded (Zeiss, Jena, Germany) scanning electron microscope (SEM) with a high-resolution FESEM with EDX, in-lens SE, SE2, BSE (EsB), and STEM detectors, and hot stage and load balancer modules at the Karabük University Iron-Steel Institute.

### 2.2. Cutting Environments

Dry, MQL, nano-MQL (graphene and hBN), Cryo, and Cryo-MQL environments were chosen as sustainable cutting environments, and a machining length of 110 mm was applied to change to new cutting inserts. MQL application was made with the Werte-STN15 lubrication system supplied by SBH Company. Cutting oil named WerteMist with a boiling point of 100 °C and a viscosity of 300 × 10^−6^ m^2^/s was sprayed at 5-bar pressure with the MQL system. The spray nozzle was located at a distance of 45 mm, and an angle of 45° to the tool tip, and the cutting fluid was sent to the cutting region at a flow speed of 100 mL/h. Cryo-cooled experiments were performed by a YDS-10 liquid nitrogen (LN_2_) tank belonging to the Lowtemp brand (Ankara, Turkey). LN_2_ was applied at 0.5-bar pressure with a 3 mm diameter nozzle.

### 2.3. Nano-MQL Preparation

**Figure 2:**
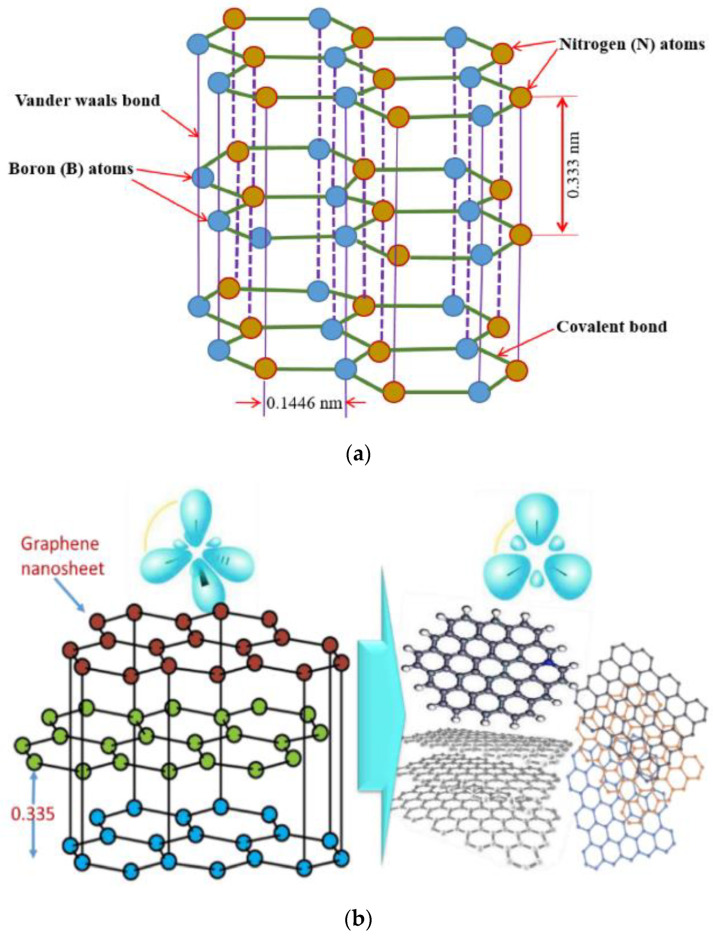
The crystal structure of, (**a**) h-BN, (**b**) graphene [49,50].

**Figure 3:**
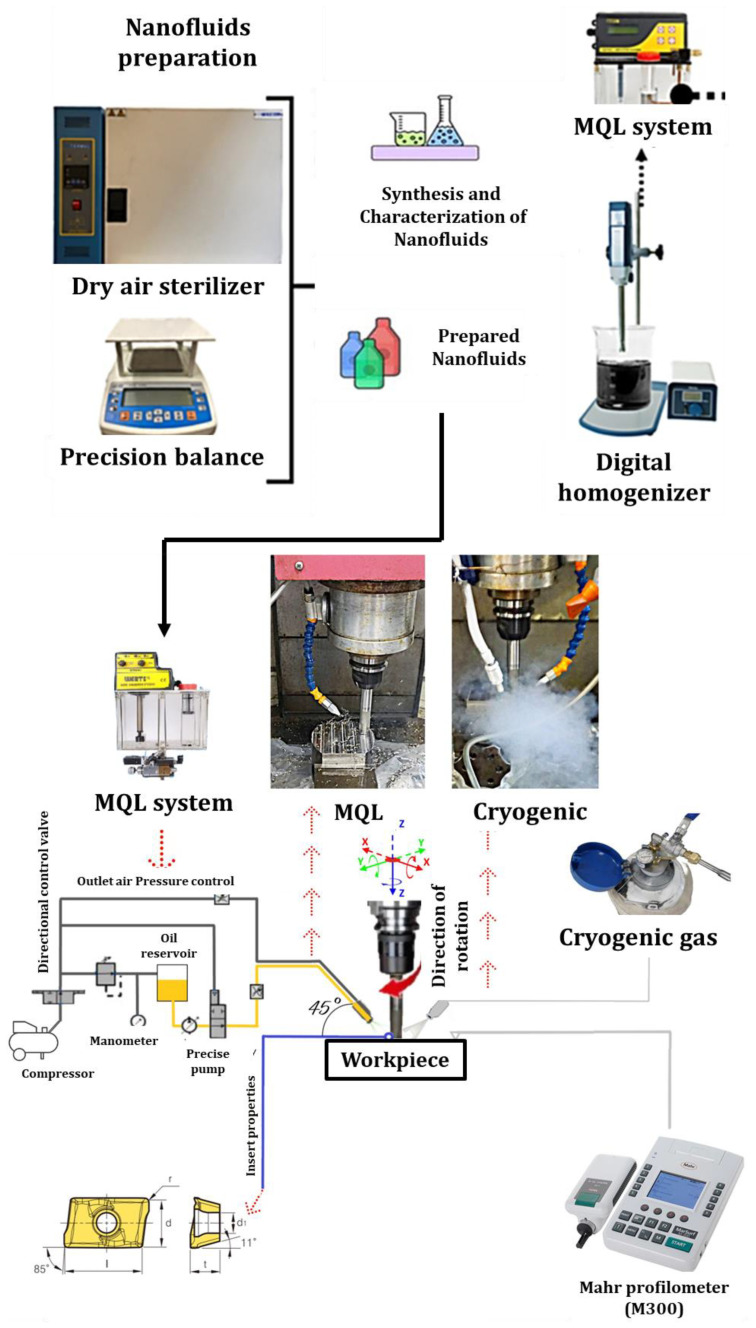
Experiment and analysis procedure.

The vegetable-based oil was supplemented with solid-state h-BN and graphene nanoparticles (Figure 2), which had an average size ranging from 40 to 50 nm. In order to achieve a uniform dispersion of nanofluid mixtures, a four-step mixing procedure was implemented. Initially, nanoparticles were introduced into the vegetable-based oil at certain volume rates (0.2% by weight) during the mixing process. During the second phase, the mixing process was carried out using a mechanical mixer operating at a speed of 300 revolutions per minute for a duration of 1 h. The third phase involved conducting the mixing process using a magnetic stirrer at a speed of 1000 rpm for a duration of 3 h. In the last stage, nanofluid mixtures exhibiting uniform dispersion of nanoparticles were obtained. In the process of nanofluid preparation, it is possible to incorporate supplementary chemical agents, such as surfactants, into the mixture to mitigate surface tension and achieve a uniform dispersion [46]. Nevertheless, the introduction of these supplementary substances can alter the characteristics of the cutting oil under elevated temperatures, impact its level of purity, and generate foam. The introduction of these substances can also result in a reduction in the thermal conductivity of the mixture due to the generation of thermal resistance [47,48]. The MQL device’s structural design effectively directs nanofluids to the cutting zone during the cooling process, resulting in the production of pressured aerosol mist vapor. In this investigation, no chemical additives were added to the nanofluid mixes due to the adverse effects of surfactants. The schematic representation of the experimental equipment and analysis technique is depicted in Figure 3.

## 3. Results and Discussion

### 3.1. Evaluation of Average Surface Roughness (Ra)

**Figure 4:**
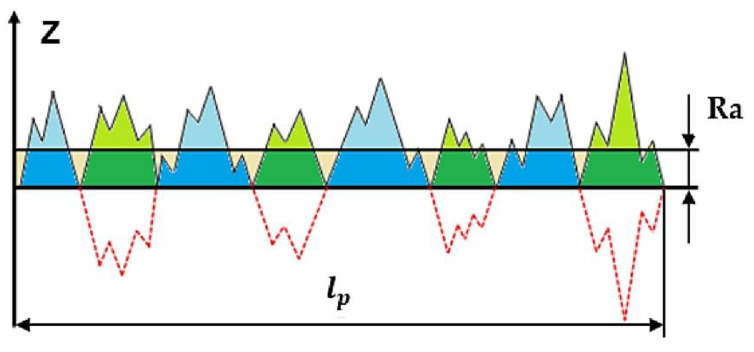
Geometric description of average surface roughness (Ra) [54].

The examination of the surface finish of the machined workpiece is of utmost importance due to its influence on the dimensional precision and mechanical characteristics of the workpiece [51,52]. Moreover, the most important surface parameter for surface integrity is the average surface roughness (Ra) [53] as schematically shown in Figure 4.

**Figure 5:**
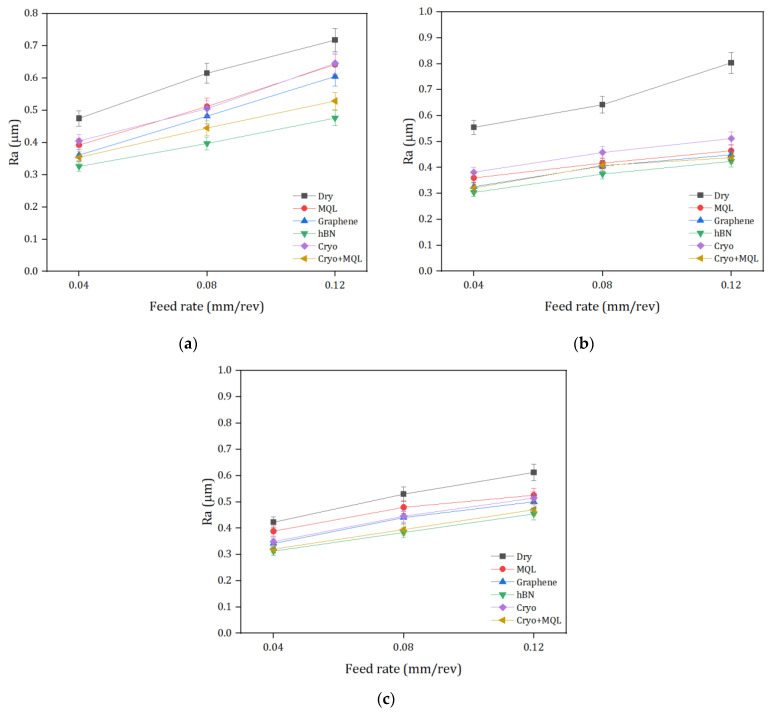
Assessment of Ra under different cooling/lubrication methods at the cutting speeds of: (**a**) 40 m/min, (**b**) 60 m/min, (**c**) 80 m/min.

The major focus of this study is the examination of surface roughness (Ra) across different machining environments, as seen in Figure 5. The results obtained from the conducted studies indicate that the dry machining technique yields the highest recorded surface roughness measurement of 0.804 µm. The subsequent processing techniques include Cryo, MQL, nMQL (graphene), Cryo-MQL, and n-MQL (hBN). The influence of comparing nanofluid with a decrease in Ra and dry to MQL may be observed. The nanofluid confining hBN exhibited enhancements of 33%, 25%, and 21% in comparison to dry, MQL, and nMQL (graphene). hBN nanoparticles possess remarkable thermal conductivity, chemical inertness, and tribological properties, rendering them a compelling candidate for use as a solid lubricant. In recent times, nanoparticles have been employed in the development of nanofluids [55]. hBN, also known as white graphite, is unaffected by the melting of metals that occurs during the cutting procedure. This can be attributed to the chemical stability and elevated melting temperature of the substance. This crystal structure has outstanding lubricating properties and possesses a low coefficient of friction. Based on these aforementioned attributes, it is expected that the system will exhibit enhanced performance under demanding machining conditions due to its increased lubrication and heat conduction properties [56]. Several studies have emphasized the performance-enhancing benefits of hBN additives in machining procedures including MQL [57,58].

Furthermore, as previously stated, the presence of nanoparticles in the lubricant mixture leads to a decrease in friction through the ball-bearing effect [59]. The capacity of nMQL to penetrate the cutting zone while providing lubrication is a crucial factor in enhancing the overall effectiveness of the substance. Furthermore, the utilization of pressured air in the MQL facilitates the seamless elimination of chips from the rake’s surface and the efficient dissipation of heat during the machining process. Furthermore, Moura et al. conducted a study that showed that the incorporation of nanoparticles into vegetable oil resulted in enhanced surface characteristics of the oil [60]. Moreover, it has been posited that the utilization of nanofluids enables the enhancement of surface quality, hence resulting in an amelioration of heat dissipation and cooling/lubricating characteristics. It is imperative to acknowledge that the incorporation of nanoparticles into a base fluid at a certain concentration has similar significance. However, it is crucial to acknowledge that the heightened concentration also impacts the lubricant mixture’s capacity to permeate [61]. Previous research has also demonstrated that exceeding a specific threshold of nanoparticle incorporation leads to a negative consequence, such as the formation of clusters at the interfaces of the machining process. This has been determined to be true [62]. Therefore, it is crucial to determine an appropriate nanoparticle concentration by evaluating thermo-physical characteristics [63].

On the other hand, nMQL (hBN) presented improvements of 26% and 10%, respectively, compared to Cryo and Cryo-MQL conditions. The improved outcomes found in nano-doped cutting oil, as compared to cryogenic cooling approaches, may be attributed to the lubricating properties of the cutting oil. The manifestation of this problem has been more apparent since the advent of solid lubricants. Solid lubricants have a stratified composition that functions as a lubricating agent. The aforementioned structure is associated with the inherent lubricating properties exhibited by solid lubricants. Even under elevated temperatures, the aforementioned structure is accountable for generating these characteristics. Furthermore, when comparing cryogenic cooling to cutting oils, it is seen that cutting oils exhibit a greater level of wettability. This characteristic leads to the formation of a film layer, hence reducing friction [64,65]. The overall results show that nano-MQL with hBN particles which are safe for operators provide the best surface quality. This approach can be considered a more sustainable method than the other lubrication and cryogenic methods since even a smaller amount of hBN (~0.2% by weight) nanoparticles increases the cooling and lubrication capacity more than the others, and it is also a cost-effective process.

### 3.2. Evaluation of Maximum Height of Surface Profile (Rz)

**Figure 6:**
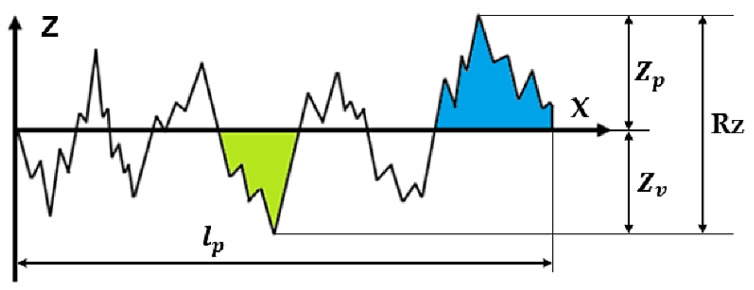
Geometric description of maximum height of surface profile (Rz) [54].

The other important parameter of surface integrity is the maximum height of the surface profile (Rz). For that reason, the study also focuses on the analysis of Rz in various machining situations, as shown in Figure 6.

**Figure 7:**
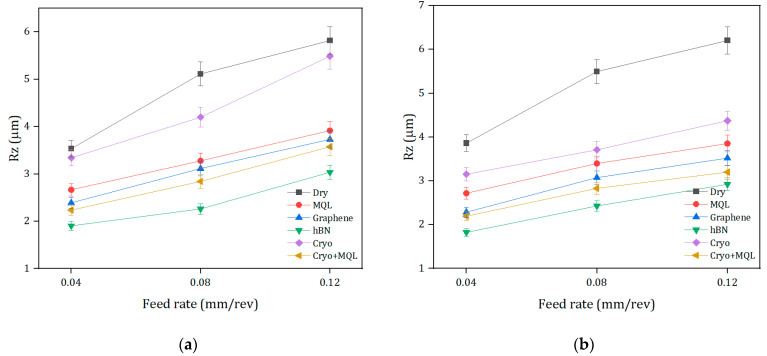

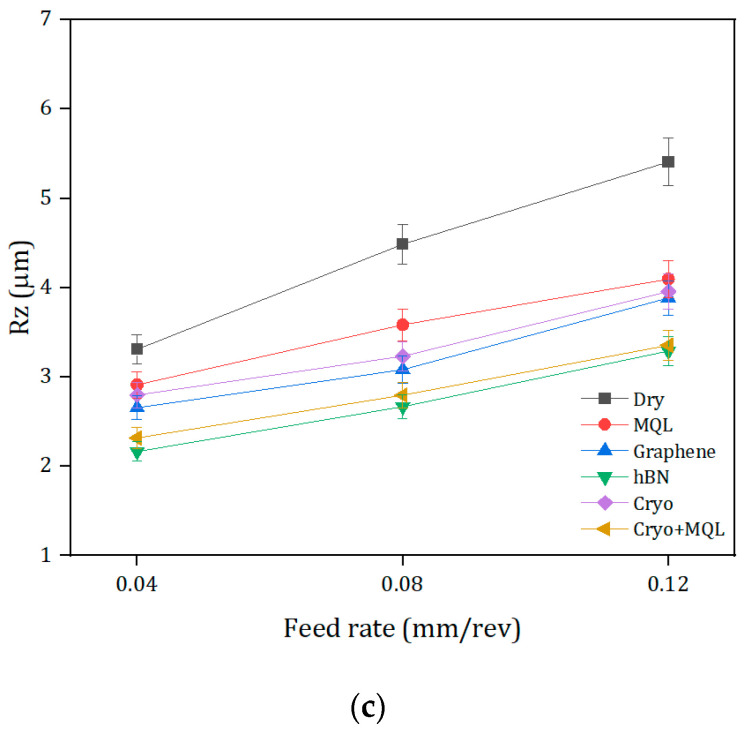
Assessment of Rz under different cooling/lubrication methods at the cutting speeds of: (**a**) 40 m/min, (**b**) 60 m/min, (**c**) 80 m/min.

This study also aims at the analysis of Rz in various machining situations as shown in Figure 7. From the experimental results, the dry machining process has the maximum Rz measurement of 6.204 µm, followed by Cryo, MQL, n-MQL (graphene), Cryo-MQL, and n-MQL (hBN). The comparison between nanofluids with a decrease in Ra and dry and MQL reveals the observed influence. Compared to dry, MQL, and n-MQL (graphene), the nanofluid containing hBN exhibited improvements of 52%, 24%, and 17%, respectively. On the other hand, nMQL (hBN) presented improvements of 33% and 9%, respectively, compared to Cryo, and Cryo-MQL conditions. The effectiveness of nMQL can be attributed to its ability to penetrate the cutting zone with lubrication. Moreover, the improved outcomes seen in nano-doped cutting oil compared to cryogenic cooling processes can be accredited to the lubrication superiority of the lubricants as also stated by Ra previously [64,65].

### 3.3. Surface Morphology

**Figure 8:**
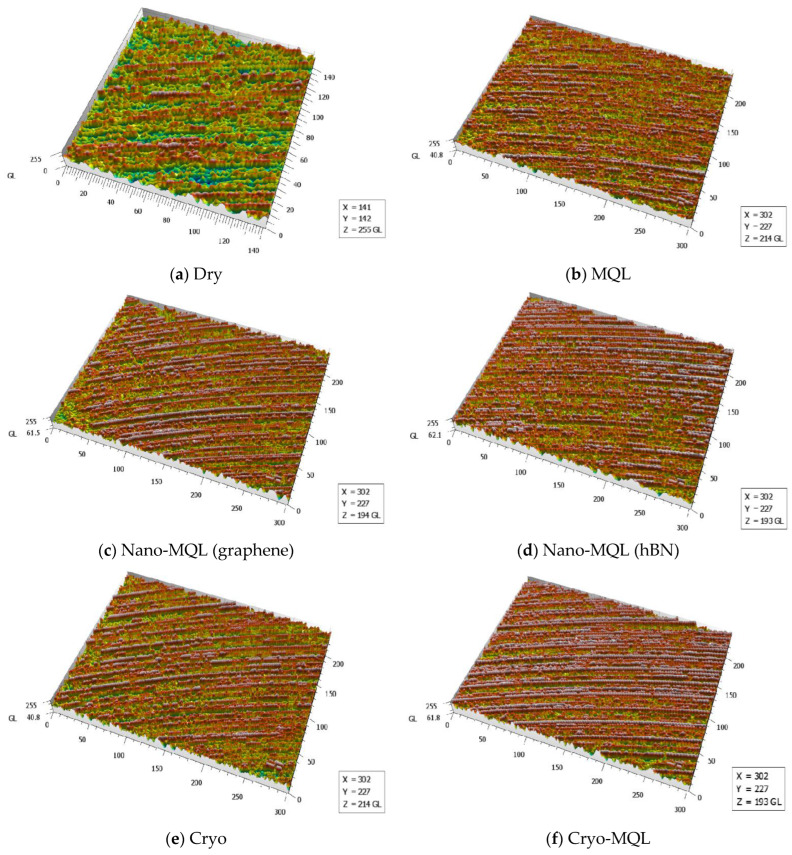
Surface morphology under different cutting environments.

The analysis and presentation of surface images generated under certain machining circumstances are likewise depicted in Figure 8. The surface finish obtained during machining determines the tribological parameters of the machined workpiece [66,67]. Surfaces generated with dry machining exhibit elevated levels of machining marks and scratches [68,69]. As anticipated, the process of dry machining led to significant heat production in the absence of cooling and lubrication. The offered comparison confirms that rapid tool wear led to higher Ra and Rz values. It has been found that the utilization of nanofluid-MQL (consisting of graphene and hBN) resulted in a significant reduction in machining marks on the surface of the workpiece, leading to the production of a smoother surface in comparison to both dry and MQL conditions. The layered structure of graphene and hBN is responsible for this phenomenon. The presence of these sliding layers decreases friction, resulting in improved surface topography. Şirin and Kıvak [70] made similar discoveries while utilizing different nanoparticles to perform the machining of nickel-based superalloy. They found that these materials resulted in improved surface topographies when nanofluids were applied. In addition, the utilization of nanofluid-MQL resulted in the effective cooling effect of MQL, which prevented the work material from sticking, simplified the chip removal, and decreased the friction, resulting in a higher surface quality [71].

Nevertheless, the application of nanoadditives coated with cutting oil and their subsequent injection into nanofluids containing hBN nanoplatelets results in the generation of a thin layer of fog that is atomized along the nozzle. This phenomenon stands in contrast to the preceding events. Consequently, it may rapidly reach the interface between the tool and the workpiece, leading to a significant enhancement in tribological properties by forming a thin film layer at that specific location [72]. During the hard-turning process, the resulting surface exhibits an increased level of anisotropy. The anisotropic-turned layer is characterized by its ability to exhibit a symmetrical and regular arrangement of peaks and valleys, setting it apart from the other layers. The evaluation of surface topography has significant importance due to its direct impact on the performance of component structures, including factors such as friction, wear, fatigue, and sealing [73]. The studies employing cryogenic freezing demonstrated a notable increase in surface stability compared to the dry condition, as seen in Figure 8. The correlation between surface quality and lubrication is evident in this specific occurrence, providing compelling proof of this relationship. The unique cooling system, referred to as cryogenic cooling, is characterized by its dual functionality of cooling and restricted lubrication. The surfaces created by cryogenic cooling exhibited a higher level of irregularity, roughness, and worse quality in comparison to those obtained using nanofluid methods. Research has demonstrated that the use of lubrication significantly mitigates the occurrence of cracks, excessive feed lines, and residue on the treated surface [74]. The investigations performed utilizing nanofluid revealed a more consistent peak-to-valley height. Conversely, it was discovered that the surfaces resulting from cryogenic cooling exhibited more complexity, irregularity, and surface damage.

### 3.4. Chip Morphology

**Figure 9:**
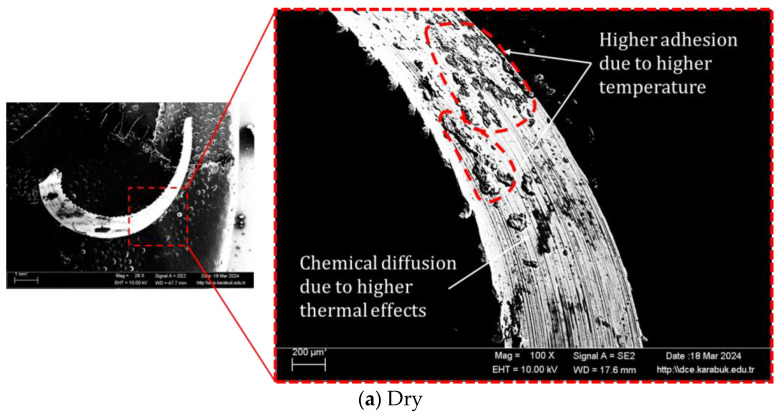

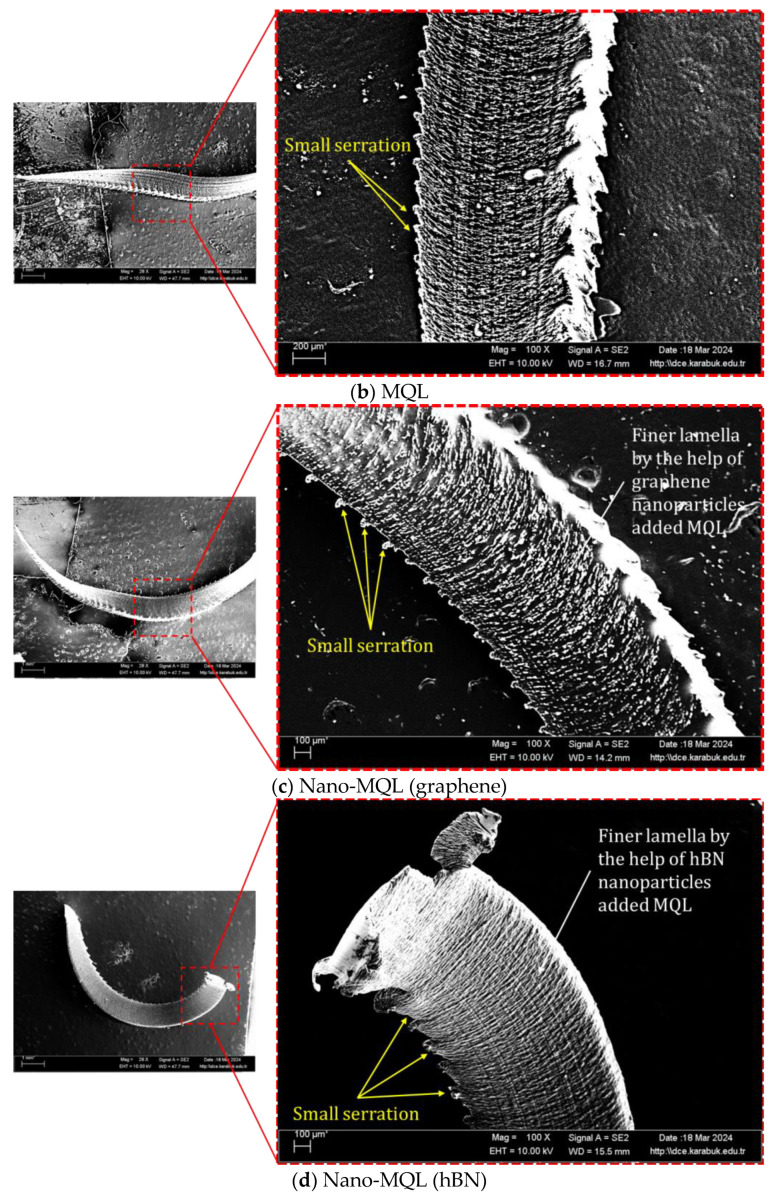

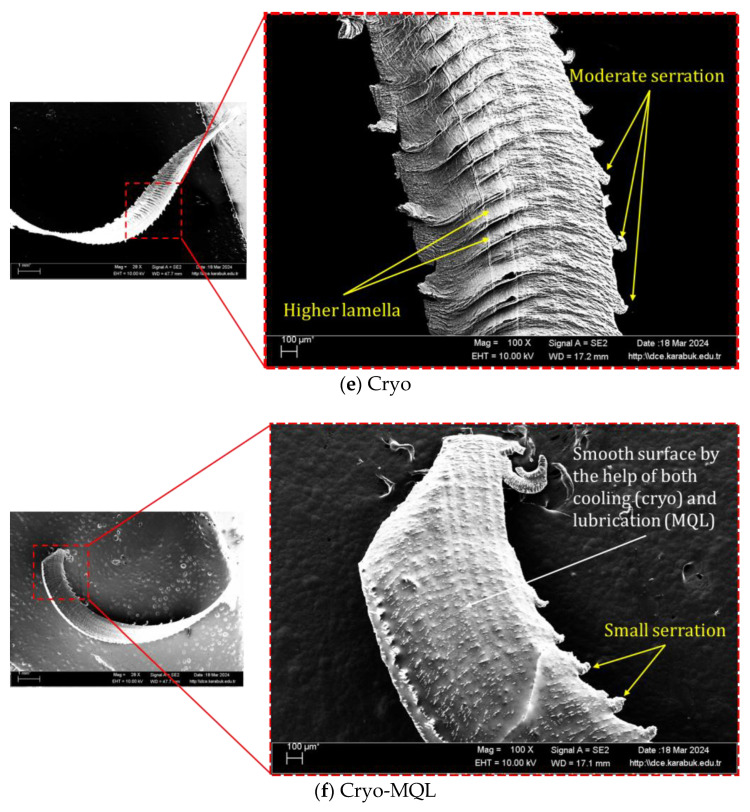
SEM images via STEM detectors for chip morphology under different cutting environments.

Significant changes in chip morphology (color, shape, serration, etc.) reflect the quality of operations in machinability as also stated by Kouam et al. [75]. Obtaining minimal tool wear and good surface quality seems likely to be directly related to chip morphology [76,77]. Another issue regarding the structure of chips is their ability to dissipate heat from the cutting zone. Regular chip formation is important for better surface quality and minimum tool wear on the processed parts [78]. Cutting parameters for regular chip formation can include the chip removal mechanism, cutting geometry, and cooling/lubrication strategies. Therefore, it is necessary to determine the chip properties in order to determine the effect of all these effective parameters. Figure 9 shows SEM images displaying the influences of various cooling and lubrication environments on chip morphology. It has been seen in the studies carried out by Şap [8] that cutting speeds do not have a visible effect on chip morphology. For this reason, only chips taken from different cooling/lubrication environments were examined. It can be seen that the chips resulting from machining in a dry environment are larger and flatter than the chips formed during machining with MQL. It was observed that the chips formed in the MQL environment were smaller and more curved. The color of chips formed in a dry environment looks different than other chips due to higher temperatures. It can be said that the chips formed in the MQL environment fall into the group of chips defined as spontaneous breaking. This situation can be called a good environment from a tribological point of view [79]. The investigation of chip shapes generated during the process of machining is of utmost importance as it offers valuable perceptions of the underlying mechanism of chip formation. The attributes of the chip removed during the machining process serve as indicators of the impact of cooling and lubrication, hence providing insights into the quality of the machining process [80]. The chip morphology is influenced by the materials used for the tool and workpiece, the circumstances under which the machining takes place, and the parameters applied for cooling and lubrication, which mostly impact the chip morphology [81]. Multiple studies have documented the examination of chip morphology in relation to different cooling and lubrication settings [82]. In the context of machining, there is a preference for shorter and fragmented chips due to their potential to decrease surface roughness. The study examines the structures of the chips generated inside the machining settings under consideration, as seen in Figure 9.

The softening of the material in dry machining, caused by increased heat generation, can be attributed to the formation of tangled, continuous, and irregular chips. Dry machining leads to a lack of cooling, resulting in increased contact duration and friction between the chip and the tool. This ultimately leads to the uneven distribution of chips. The MQL process involves the generation of segmented fragmented chips, whereas the nMQL machining process demonstrates a decrease in the number of contact zones between the chip and the tool. The observed reduction in the curvature radius of the chips generated suggests that the use of nMQL led to enhanced lubrication. The results align with earlier research, indicating that chips with a reduced curling radius exhibit superior penetration and lubrication when combined with MQL and nMQL. This observation is consistent with the aforementioned results [83]. Darshan et al. [84] also observed comparable outcomes while employing continuous helical chips in dry machining, as well as tiny fragmented chips with hBN-aided MQL. These findings indicated improved lubrication and a decreased likelihood of built-up edge development. Moreover, an analysis is conducted on the chip morphology generated under various machining settings. The production of serrated chips is observed across various machining settings, as depicted in Figure 9. Under dry machining, the chips exhibited maximal serrations due to increased friction between the chip and tool contact and plastic deformation (Figure 9a). Conversely, research has demonstrated that chips with a small degree of serration had improved heat transfer from the machining area and reduced frictional impacts at the interface between the chip and the tool when exposed to MQL and nMQL. The chip surface was broken by the MQL application, resulting in the formation of an uneven lamella due to increased friction, as seen in Figure 9b. The utilization of MQL and nanoparticles has been seen to have a substantial effect on the regulation of friction at the interface between the chip and the tool. Chips exhibiting a fine lamella structure are generated with the utilization of nano-MQL (as seen in Figure 9c,d), owing to the concurrent decrease in friction and temperature. The application of nMQL results in enhanced chip morphology due to the efficient penetration of cutting fluid containing nanoparticles between chip–tool contacts, leading to a reduction in friction. Airao et al. [85] observed comparable findings in their study on conventional turning conducted under various circumstances, including dry, wet, MQL, and LCO2. In the context of dry machining, chips exhibiting non-uniform fractures were acquired, while chips with cracked surfaces were detected during MQL conditions.

However, the chip surfaces exhibit a higher degree of smoothness in the experimental trials that incorporate cryogenic cooling. The chip notch tooth rate exhibits an upward trend with increasing cutting speed in cryogenic cooling, indicating a higher level of efficiency compared to nanofluid (Figure 9). There exists a correlation between this particular situation and the efficacy of cryogenic cooling in delivering sufficient cooling. The utilization of cryogenic cooling, known for its efficacy in delivering efficient cooling during high-speed operations, played a pivotal role in delaying the deterioration of the cutting tool and facilitating the successful culmination of the cutting procedure. Prior studies have demonstrated that this situation exhibits similarities. Bermingham et al. [86] posited that the utilization of cryogenic cooling had favorable outcomes in terms of chip morphology. Ross et al. [87] found that cryogenic cooling improved the chip’s ability to break. The operational characteristics of cooling systems differ according to their inherent nature. In the case of nanofluid, the spray pressure was measured to be 8 bar, but the spray pressure for LN2 was found to be 15 bar. The chip’s susceptibility to breakage may have been heightened due to the application of elevated pressure within the cryogenic system, perhaps yielding a marginally advantageous outcome.

## 4. Conclusions and Recommendations

The study conducted an analysis of the alterations in chip morphology, surface morphology, and surface quality criteria (Ra and Rz) that took place during the milling process of precipitation-hardened steel (PH13-8Mo) under various cutting conditions. The milling studies were conducted in several settings, including dry, MQL, nano-MQL (graphene), nano-MQL (hBN), Cryo, and Cryo-MQL. TiAlN-coated inserts were used, and three different cutting speeds and feed rates were employed. The summarized results are indicated below:

- Comparisons between nanofluid with reduced Ra and dry and MQL show that the nanofluid with hBN outperformed dry, MQL, and nano-MQL (graphene) by 33%, 25%, and 21%, respectively. Moreover, nano-MQL (hBN) improved by 26% and 10% over Cryo and Cryo-MQL, respectively.
- Differences between nanofluid with reduced Rz and dry and MQL display the effect that the nanofluid with hBN outperformed dry, MQL, and nano-MQL by 52%, 24%, and 17%. Additionally, the nano-MQL (hBN) showed 33% and 9% improvements over the Cryo and Cryo-MQL conditions, respectively.
- The ability of nMQL to infiltrate the cutting zone while simultaneously providing lubrication is a critical aspect in improving the overall efficiency of the drug. In addition, the use of compressed air in the MQL system allows for the effective removal of chips from the rake face and the efficient dispersal of heat during the machining process. Furthermore, the enhanced surface quality results observed in nano-doped cutting oil, in comparison to cryogenic cooling methods, can be ascribed to the lubricating characteristics of the cutting oil. The emergence of solid lubricants has made this problem more evident. Solid lubricants have a layered structure that serves as a lubricant.
- In cryogenic cooling experiments, chip surfaces are smoother, and the chip notch tooth rate increases with the cutting speed, suggesting more efficiency than nanofluid. Cryogenic cooling’s ability to cool is attributed to the current situation. Cryogenic cooling, known for its efficiency in quick operations, was essential to keeping the cutting tool from degrading and completing the cutting.

## Figures and Tables

**Table 1:** The chemical composition of the workpiece material (wt. %).

C	Mn	Si	P	Ni	Cr	Mo	Al	N	S	Balance
0.05	0.1	0.1	0.01	8.5	13.2	2.2	0.96	0.01	0.08	Fe

**Table 2:** Cutting parameters.

Cutting Speed, m/min	Feed Rate, mm/Tooth	Cutting Depth, mm
40–60–80	0.04–0.08–0.12	0.8

## Data Availability

Data used in this work can be requested by contacting the first author.
